# Supplementary material for: Assessment of Communication Abilities in Four Children with Early Bilateral CIs in Clinical and Home Environments with LENA System: A Case Report
Source: Children (Basel). 2022 May 4;9(5):659. doi: 10.3390/children9050659 (PMC9140017; doi:10.3390/children9050659)
Supplement: Supplementary file 1 [file children-09-00659-s001.zip › children-1661693-supplementary.pdf]

Supplementary material S1.

Raw data table.

Date LENA	File Hours	ChildID	Child birthdate	Child Age LENA	AWC LENA	Turns LENA	Child.Voc LENA	EMLU LENA	Date exam	Child Age exam	AWC videoanalysis	Child.Voc videoanalysis	EMLU videoanalysis	Turns videoanalysis	Receptive vocabulary	Productive vocabulary
07/03/17	10.46	C003	22/07/16	7	2518	98	411	0.00	13/02/17	6.7	496	16	0.00	0		
16/05/17	12.71	C003	22/07/16	9	3080	52	188	0.00								
08/12/17	10.30	C003	22/07/16	16	1593	73	305	1.05	04/12/17	16.4	478	20	0.00	0	5	5
12/05/18	15.56	C003	22/07/16	21	1206	89	325	1.37	22/06/18	23	686	41	1.00	20	5	25
02/11/18	10.88	C003	22/07/16	27	2110	202	474	2.33								
03/02/19	8.67	C003	22/07/16	30	1937	162	580	2.49	23/01/19	30.03	659	108	1.28	85	25	25
12/05/19	9.26	C003	22/07/16	33	1689	165	408	2.73	05/06/19	34.43	469	72	1.42	80	50	50
18/07/19	11.16	C003	22/07/16	35	2727	159	430	3.13								
09/04/17	10.23	C004	15/09/16	6	3564	101	205	0.00								
27/05/17	10.14	C004	15/09/16	8	2724	92	212	0.00	11/07/17	9.87	533	70	0.00	0	25	50
22/09/17	10.32	C004	15/09/16	12	3396	101	277	0.00	21/09/17	12.2	640	102	0.00	0		
30/12/17	10.54	C004	15/09/16	15	2303	84	241	1.05	20/11/17	17.17	650	85	1.00	55	25	25
26/04/18	10.43	C004	15/09/16	19	2131	89	201	0.00	20/02/18						50	25
05/08/18	10.07	C004	15/09/16	22	3704	186	278	1.53	21/08/18	23.2	858	109	1.03	65	50	50
01/11/18	10.32	C004	15/09/16	25	1562	125	446	1.93	23/11/18	26.27				90	75	50
17/02/19	10.37	C004	15/09/16	29	846	38	174	2.09								
20/05/19	10.64	C004	15/09/16	32	1458	92	422	3.05	15/05/19	32				40	75	50
29/07/19	10.36	C004	15/09/16	34	1036	62	255	3.21								
28/09/19	10.61	C004	15/09/16	36	1256	45	341	3.13	15/11/19						95	95
06/04/17	12.65	C005	16/09/16	6	2105	87	301	0.00								
19/06/17	11.31	C005	16/09/16	9	1219	57	358	0.00	18/07/17	10.07	225	8	0.00	5		
02/10/17	12.79	C005	16/09/16	12	2503	105	361	0.00	18/08/17	11.07	306	11	0.00	5		
16/12/17	11.55	C005	16/09/16	15	2104	113	336	1.13	24/01/18	16.27	490	40	1.00	40	25	25
15/04/18	16.00	C005	16/09/16	18	2217	117	381	1.05	24/08/18							25
21/10/18	11.41	C005	16/09/16	25	3344	245	637	1.93	30/10/18	23.17				85	90	50
20/01/19	10.36	C005	16/09/16	28	1600	142	497	2.25		25.47	615	63	1.19	75	90	
07/04/19	12.66	C005	16/09/16	30	2243	150	606	2.41	11/04/19	30.83	459	62	1.69	75		
02/08/19	12.61	C005	16/09/16	34	2056	169	826	2.89	04/07/19	33.6	634	100	2.17	80	90	50
06/10/19	9.02	C005	16/09/16	36	3308	234	545	2.89	18/09/19					85		
11/04/17	10.51	C006	01/01/16	15	2713	131	330	0.00	13/02/17	13.4	488	79	0.00	5		
11/07/17	10.41	C006	01/01/16	18	2386	132	464	0.00	19/07/17	18.6	367	58	0.00	20	10	25
22/10/17	14.03	C006	01/01/16	21	2303	146	460	1.29	22/09/17	20.7	487	43	1.00	20		
13/01/18	11.65	C006	01/01/16	24	2667	152	489	1.53	29/01/18	24.93	509	80	1.33	25	5	5
22/04/18	16.00	C006	01/01/16	27	4823	193	485	1.85								
02/11/18	6.86	C006	01/01/16	34	1300	56	316	2.33	16/10/18	33.5	535	70	1.47	75	50	5
29/01/19	4.02	C006	01/01/16	36	2975	122	469	2.57	19/04/19						75	95

Supplementary material S2.

Multiple correlation plots for each child separately. The heat bar indicates the size of the correlation coefficient, whereas the stars indicate significance value <= 0.05.

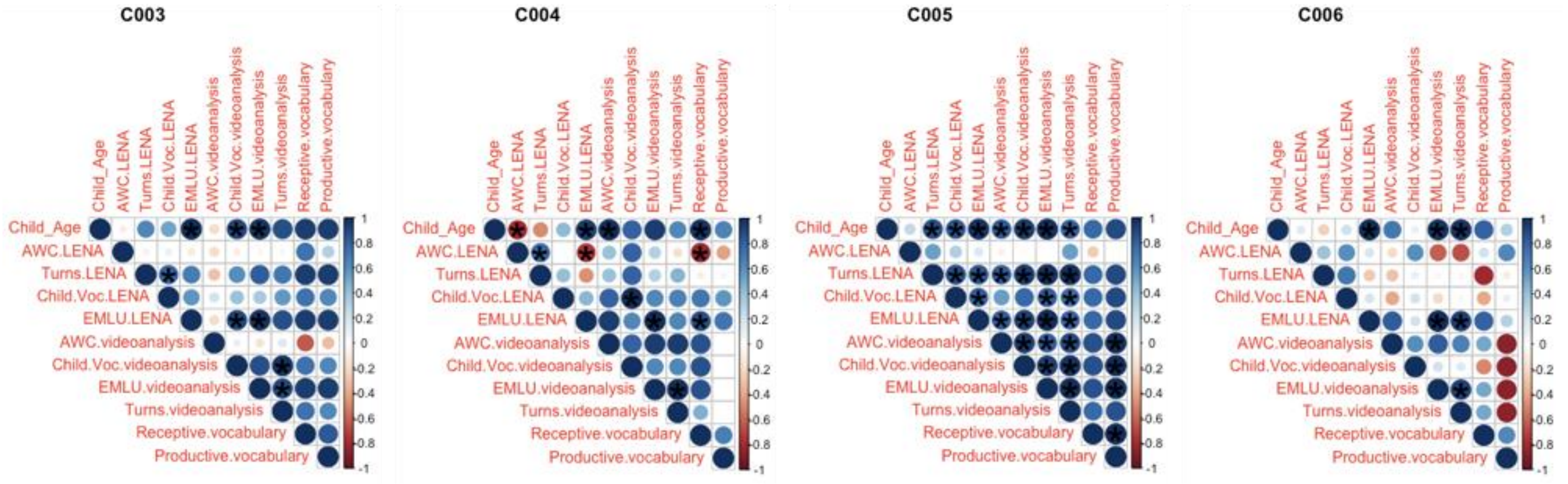

Supplementary material S3
The predictors of Productive vocabulary
mp: Productive.vocabulary ~ Turns.videoanalysis + EMLU.LENA + EMLU.videoanalysis + Child_Age + (Child_Age|ChildID)
Data: raw data
Linear mixed model fit by REML. t-tests use Satterthwaite's method ['lmerModLmerTest']
REML criterion at convergence: 76.7

Scaled residuals:

Min	1Q	Median	3Q	Max
−0.68	−0.10	−0.02	0.07	0.67

Random effects:

Groups	Name	Variance	Std.Dev.	Corr
ChildID	(Intercept)	2845.1	53.34	
	Child_Age	2.34	1.53	−0.70
Residual		2.38	1.54	

Number of obs: 15, groups: ChildID, 4

Fixed effects:

	Estimate	Std.Error	df	t value	Pr(>|t|)	
(Intercept)	−45.37	26.94	3.07	−1.68	0.19	
Turns.videoanalysis	−0.69	0.06	0.97	−12.17	0.06	.
EMLU.LENA	−33.05	3.52	0.96	−9.38	0.07	.
EMLU.videoanalysis	−1.64	2.43	0.95	−0.67	0.63	
Child_Age	7.04	0.85	3.93	8.23	0.001	**

Type III Analysis of Variance Table with Satterthwaite's method

	SumSq	MeanSq	NumDF	DenDF	F-value	Pr(>F)	
Turns.videoanalysis	352.90	352.90	1	0.97	148.15	0.06	.
EMLU.LENA	209.76	209.76	1	0.96	88.06	0.07	.
EMLU.videoanalysis	1.08	1.08	1	0.96	0.46	0.6	
Child_Age	161.43	161.43	1	3.93	67.77	0.001	**

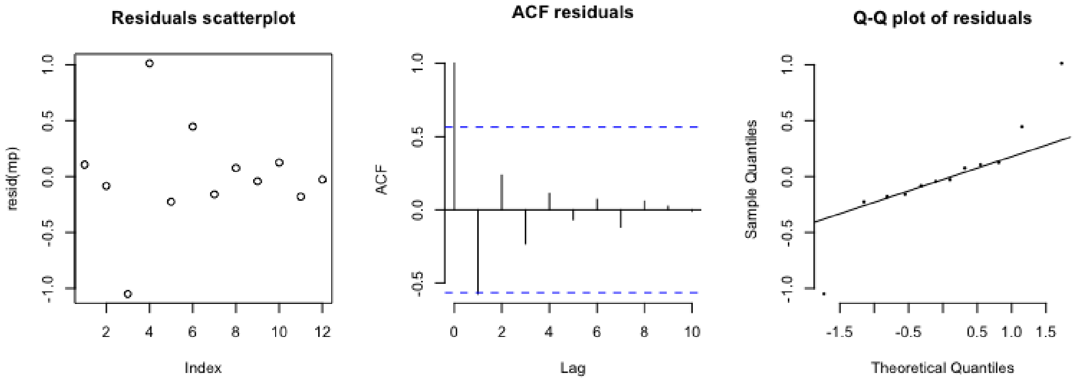

Figure S3-1 Plots of conditional residuals for the model mp (a scatterplot, autocorrelation, and the plot of the quantiles' distribution).

The predictors of Receptive vocabulary

mr: Receptive.vocabulary ~ Turns.videoanalysis + EMLU.LENA + EMLU.videoanalysis + Child_Age + (Child_Age|ChildID);
Data: raw data
Linear mixed model fit by REML. t-tests use Satterthwaite's method ['lmerModLmerTest']
REML criterion at convergence: 81

Scaled residuals:

Min	1Q	Median	3Q	Max
−1.50	−0.42	0.09	0.56	0.81

Random effects:

Groups	Name	Variance	Std.Dev	Corr
ChildID	(Intercept)	1.05e+03	32.42	
	Child_Age	7.14e-02	0.27	−0.20
Residual		4.33e+01	6.58	

Number of obs: 13, groups: ChildID, 4

Fixed effects:

	Estimate	Std.Error	df	t-value	Pr(>|t|)	
(Intercept)	−42.9	19.54	3.72	−2.2	0.1	.
EMLU.LENA	−0.29	11.88	5.98	−0.02	0.98	
EMLU.videoanalysis	−26.5	8.3	4.11	−3.19	0.03	*
Child_Age	4.64	1.06	6.23	4.36	0.004	**

Type III Analysis of Variance Table with Satterthwaite's method

	SumSq	MeanSq	NumDF	DenDF	F-value	Pr(>F)	
EMLU.LENA	0.03	0.03	1	5.98	0.001	0.98	
EMLU.videoanalysis	441.89	441.89	1	4.11	10.2	0.03	*
Child_Age	822.08	822.08	1	6.23	18.98	0.004	**

Figure S3-2 Plots of conditional residuals for the model mr (a scatterplot, autocorrelation, and the plot of the quantiles' distribution).

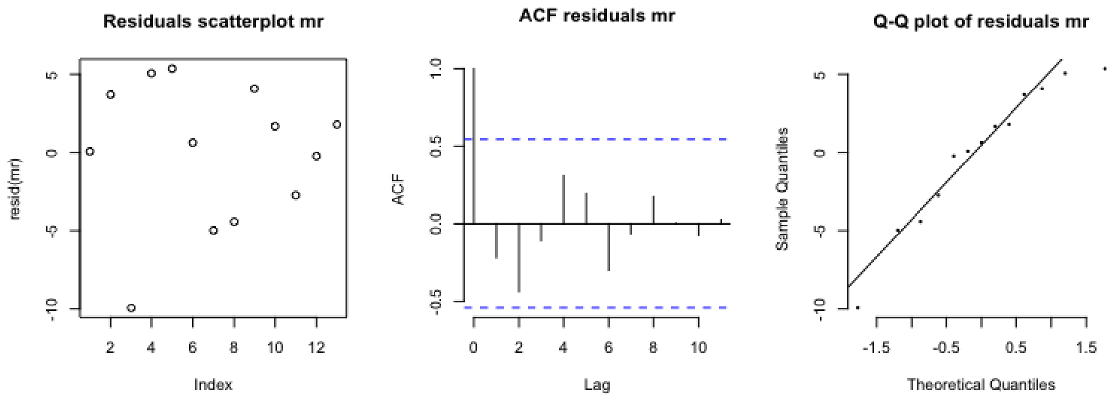
Residuals scatterplot mr
resid(mr)
Index
5
0
-5
-10
2
4
6
8
10
12
ACF residuals mr
ACF
Lag
1.0
0.5
0.0
-0.5
0
2
4
6
8
10
Q-Q plot of residuals mr
Sample Quantiles
Theoretical Quantiles
5
0
-5
-10
-1.5
-0.5
0.5
1.5

Supplementary material S4

===

FOGLIO INFORMATIVO E MODULO DI CONSENSO INFORMATO
PER LA PARTECIPAZIONE ALLO STUDIO

===

TITOLO DELLO STUDIO: **Valutazione delle capacità di comunicazione in bambini con primi IC bilaterali in ambienti clinici e domestici: 4 relazioni di casi**
CODICE DEL PROTOCOLLO: RC n. 17/17
PROMOTORE: IRCCS materno infantile "Burlo Garofolo"
STRUTTURA PRESSO LA QUALE SI SVOLGERÀ LO STUDIO: S.C. Otorinolaringoiatria e audiologia
SPERIMENTATORE RESPONSABILE: dott. Orzan Eva
CONTATTI per lo SPERIMENTATORE RESPONSABILE: Referente scientifico Dott.ssa Amanda Saksida
Tel: +386.40.843378 E-mail: amanda.saksida@burlo.trieste.it

Gentili genitori,
con il presente modulo invitiamo Vostro/a figlio/a a partecipare allo studio no profit dal titolo "*Valutazione delle capacità di comunicazione in bambini con primi IC bilaterali in ambienti clinici e domestici: 4 relazioni di casi*" promosso dalla S.C. Otorinolaringoiatria e audiologia, IRCCS materno infantile "Burlo Garofolo" di Trieste.
Lo sperimentatore principale (PI) dello studio è la dott.ssa Eva Orzan (tel. 040.3785.318. E-mail: eva.orzan@burlo.trieste.it), direttore della S.C. Otorinolaringoiatrai e audiologia, coadiuvato dalla dr.ssa Amanda Saksida, afferente alla medesima struttura.
La partecipazione allo studio è a carattere volontario; avete a disposizione un tempo adeguato per riflettere e porre domande di chiarimento prima di dare la propria adesione. Come genitori del soggetto coinvolto nello studio avete il diritto di ritirare il Vostro consenso in qualsiasi momento senza dover fornire alcuna giustificazione senza perdere alcun diritto e beneficio. In caso di ritiro del consenso nessuna nuova informazione sarà raccolta e aggiunta ai dati esistenti. Come genitori del soggetto avete il diritto di sapere ogni nuova informazione che possa modificare la vostra decisione di partecipare allo studio.

Obiettivo dello studio
<u>Lo studio ha lo scopo di:</u>

- Valutare le misure cliniche dello sviluppo del linguaggio e le misure delle videoanalisi in confronto con le analisi automatiche delle registrazioni giornaliere del ambiente sonore del bambino, ottenute attraverso il sistema LENA e effettuate a casa dei partecipanti.

Caratteristiche dello studio:
La partecipazione allo studio viene proposta a 4 bambini portatori di impianto cocleare bilaterale precoce di età compresa tra i 6 ed i 36 mesi, afferenti alla S.C. Otorinolaringoiatria e audiologia dell'IRCCS Burlo Garofolo. Le registrazioni con LENA vengono proposti ogni tre mesi nel periodo da 6 a 36 mesi d'eta. Le registrazioni vengono effettuate a casa e somministrate dai genitori dei partecipanti.
I dati delle registrazioni giornaliere a casa vengono analizzate assieme alle misure cliniche dello sviluppo del linguaggio e le misure delle videoanalisi, ottenute nello stesso periodo delle registrazioni giornaliere.

Rischi ed effetti collaterali che possono derivare dalla partecipazione allo studio
Le procedure previste non comportano alcun rischio o effetto collaterale.

Possibili Benefici derivanti dalla partecipazione allo studio
In considerazione del carattere innovativo di questo studio, non sono allo stato attuale prevedibili benefici diretti per chi vi partecipa. Quella che va comunque sottolineata è l'assenza di nocività della tecnica usata nella ricerca.

Presenza della copertura assicurativa ed (solo se pertinenti) eventuali modalità della copertura ai sensi del D.M. 14.07.2009
La polizza assicurativa Aziendale copre i danni derivanti dalla partecipazione allo studio e relativa copertura del rischio postumo.

Diritti del paziente (compreso il diritto di ritirare in qualsiasi momento il consenso alla partecipazione allo studio)
Vi e garantito un tempo sufficiente di riflessione e consultazione, e di porre domande di chiarimento; l'adesione alla partecipazione allo studio sarà volontaria. In qualsiasi momento avete la possibilità di ritirare il consenso senza addurre alcuna motivazione e senza che Vostro/a figlio/a subisca alcuna conseguenza o perdita dei benefici. Inoltre la partecipazione allo studio è gratuita. Dal momento che lo studio non prevede costi data la sua natura osservazionale non sono previsti indennizzi di alcun genere per i pazienti.

È responsabilità del paziente: Si precisa peraltro che il paziente avrà diritto ad ottenere il risarcimento di eventuali danni da parte del responsabile del danno stesso nei termini previsti dalle leggi vigenti.
Se, in qualità di rappresentanti legali, decidete di partecipare allo studio, vi chiederemo inoltre manifestare il consenso al trattamento dei dati personali. L'informativa ed il consenso al trattamento dei dati personali vanno tenuti distinti da informativa e consenso alla partecipazione allo studio e saranno predisposti secondo la normativa sulla privacy attualmente in vigore. Il rifiuto a conferire i dati personali non consentirà di partecipare allo studio.

Cosa si prevede se il paziente decide di non partecipare allo studio
Il minore che rappresentate godrà del miglior trattamento al momento disponibile.

Normativa di riferimento
Tutte le informazioni raccolte durante lo studio sono confidenziali e verranno trattate in ottemperanza al D. Lgs. 196 del 30 Giugno 2003 "Codice in materia di protezione dei dati personali" e alla Deliberazione n. 52 del 24.07.2008 "Linee Guida per il trattamento dei dati

nell’ambito delle sperimentazioni cliniche di medicinali”, del Garante per la Privacy. Il rifiuto di conferire i dati personali non consente la partecipazione allo studio.
Nel caso desideraste ricevere ulteriori chiarimenti o informazioni potete rivolgersi a:
Dr. Amanda Saksida presso la S.C. Otorinolaringoiatria ed Audiologia, tel. 040.3785.218 – e-mail: amand.saksida@burlo.trieste.it

Possibilità per i genitori del paziente di richiedere comunicazioni e informazioni circa i risultati dello studio
Se lo desiderate, verrete informati di eventuali imprevisti e novità riguardanti lo studio e, alla fine della ricerca, potranno esservi comunicati i risultati emersi.

Altre informazioni
Se lo desiderate, potete informare il Vostro medico di famiglia (o altro medico da Voi indicato) della Vostra partecipazione allo studio, il quale potrete contattare il medico dello studio per qualsiasi informazione.

Vi informiamo anche che il protocollo dello studio che Vi è stato proposto è stato redatto in conformità alle Norme di Buona Pratica Clinica dell’Unione Europea e alla revisione corrente della Dichiarazione di Helsinki ed è stato approvato dal Comitato Etico Unico Regionale del Friuli Venezia Giulia, dalle competenti Autorità Sanitarie o dalle Istituzioni da queste delegate.

Referente dello studio
Per qualunque dubbio, domanda o richiesta di precisazioni inerenti lo studio potrete contattare: Dr. Amanda Saksida presso la S.C. Otorinolaringoiatria ed Audiologia, tel. 040.3785.218 – e-mail: amanda.saksida@burlo.trieste.it

==

MODULO DI CONSENSO INFORMATO PER LA PARTECIPAZIONE ALLO STUDIO

==

TITOLO DELLO STUDIO: **Valutazione delle capacità di comunicazione in bambini con primi IC bilaterali in ambienti clinici e domestici: 4 relazioni di casi**
CODICE DEL PROTOCOLLO: RC n. 17/17
PROMOTORE: IRCCS materno infantile "Burlo Garofolo"
STRUTTURA PRESSO LA QUALE SI SVOLGERÀ LO STUDIO: S.C. Otorinolaringoiatria e audiologia
SPERIMENTATORE RESPONSABILE: dott. Orzan Eva
CONTATTI per lo SPERIMENTATORE RESPONSABILE: Referente scient. Dott.ssa Amanda Saksida. Tel: +386.40.843378 E-mail: amanda.saksida@burlo.trieste.it

PAZIENTE ____________________ ________________________ *(Nome e Cognome in stampatello)*

Numero identificativo assegnato al/alla paziente: ______

Io sottoscritta (*Nome e Cognome in stampatello*) ___________________________________,

nata _________________ il: __/__/_____ (madre)

Io sottoscritto (*Nome e Cognome in stampatello*) ___________________________________,

nato _________________ il: __/__/_____ (padre)

residente in ______________________________ Prov. __________ CAP ___________

Via _____________________n° _____Tel. ___________________

in proprio, ovvero nella qualità di RAPPRESENTANTE LEGALE / AMMINISTRATORE DI SOSTEGNO di

(*Nome e Cognome in stampatello*) ____________________ ________________________,

nato/a a ____ il ____________________________

residente in ______________________________ Prov. __________ CAP ___________

Via _____________________n° _____Tel. ___________________

Resta inteso che, nel caso in cui il paziente dovesse recuperare la capacità di intendere e di volere, sarà cura dello sperimentatore ottenere il consenso scritto da parte del paziente stesso (D.L. del 21-12-07, 6.1.2.5).
In caso sia designato l'amministratore di sostegno, il medico sperimentatore avrà cura di verificare che l'ordinanza di affidamento da parte del giudice tutelare comprenda la tutela della salute dell'amministrato.

dichiaro di aver ricevuto informazioni dettagliate e comprensibili, scritte e verbali, sulla natura, il significato, i benefici attesi, i rischi, e le implicazioni dello studio. Inoltre, ho letto e compreso il testo delle informazioni per il paziente.

Ho discusso con lo sperimentatore circa la conduzione dello studio ed ho avuto la possibilità di porre domande sullo studio. Tutte le mie domande hanno avuto risposte soddisfacenti in merito ai punti non chiari.

Dichiariamo:

- di aver avuto il tempo necessario per riflettere sulle informazioni ricevute e per prendere liberamente una decisione e di aver avuto la possibilità di discuterne con lo sperimentatore, con il medico curante, con i familiari, amici, conoscenti;
- di aver compreso lo scopo di questo studio e le procedure alle quali sarò sottoposta/o;
- di essere libero/a di rifiutare di partecipare allo studio proposto, senza fornire un motivo e senza che le cure mediche o i diritti legali di nostro/a figlio/a vengano compromessi;
- di dare volontariamente il mio consenso alla partecipazione di nostro/a figlio/a allo studio, come mi è stato descritto in questo documento, senza aver avuto coercizioni e di essere consapevole della possibilità di revocare il mio consenso alla partecipazione allo studio in qualsiasi momento senza addurre alcuna motivazione (oralmente o per iscritto), senza perdere alcun diritto o beneficio, senza pregiudicare le cure mediche né i diritti legali di nostro/a figlio/a e senza che tale scelta modifichi in alcun modo i nostri rapporti con il personale medico e sanitario della struttura;
- di acconsentire a che i rappresentanti dello sponsor, il comitato etico e gli enti normativi abbiano accesso ai miei dati medici;
- di essere consapevole che i risultati dello studio saranno resi noti alla comunità scientifica e che l'identità di nostro/a figlio/a non sarà menzionata in nessun resoconto dello studio e che tutte le informazioni ottenute nel corso della sperimentazione saranno trattate come strettamente confidenziali;
- di dare il nostro consenso all'acquisizione, elaborazione, utilizzo, analisi e comunicazione in alcune pubblicazioni dei dati sensibili di nostro/a figlio/a codificati pertinenti raccolti durante lo studio e delle informazioni sulla sua salute, adeguatamente elaborate in forma anonima.

Comprendiamo inoltre che riceveremo una copia di questo documento da noi firmato. L'originale rimarrà presso il centro della sperimentazione. Questo consenso è valido a meno che e fino a quando noi non lo revocheremo.
Acconsentiamo inoltre a:

- rendere disponibili i dati personali riservati per le procedure di controllo della qualità e per le ispezioni da parte delle autorità/istituzioni competenti e da parte del Comitato Etico;
- informare il medico responsabile dello studio nel caso in cui decida di ritirarmi dallo studio.

◎ ACCONSENTIAMO - NON ACCONSENTIAMO: a partecipare allo studio sopra indicato.

◎ ACCONSENTIAMO - NON ACCONSENTIAMO: a che lo sperimentatore responsabile informi il mio medico di medicina generale (o altro medico da me indicato) della mia partecipazione a questo studio.
◎ ACCONSENTIAMO - NON ACCONSENTIAMO: ad essere informato/a dal medico responsabile sui risultati di questo studio.
Dichiarazioni eventuali dei genitori domande aggiuntive o altri aspetti del consenso informato:

____

Nome e cognome per esteso del/la paziente o del legale rappresentante / amministratore di sostegno in stampatello:
(madre)____
(padre)____
Data: ________________
Firma del/la paziente o del rappresentante legale / amministratore di sostegno:
(madre)____
(padre)____

==

INFORMATIVA E MANIFESTAZIONE DEL CONSENSO AL TRATTAMENTO DEI DATI PERSONALI

==

TITOLO DELLO STUDIO: **Valutazione delle capacità di comunicazione in bambini con primi IC bilaterali in ambienti clinici e domestici: 4 relazioni di casi**<br>CODICE DEL PROTOCOLLO: RC n. 17/17<br>PROMOTORE: IRCCS materno infantile "Burlo Garofolo"<br>STRUTTURA PRESSO LA QUALE SI SVOLGERÀ LO STUDIO: S.C. Otorinolaringoiatria e audiologia<br>SPERIMENTATORE RESPONSABILE: dott. Orzan Eva<br>CONTATTI per lo SPERIMENTATORE RESPONSABILE: Referente scientifico Dott.ssa Amanda Saksida<br>Tel: +386.40.843378 E-mail: amanda.saksida@burlo.trieste.it

Gentili genitori,

Avete accettato di far partecipare Vostro/a Figlio/a allo "*Valutazione delle capacità di comunicazione in bambini con primi IC bilaterali in ambienti clinici e domestici: 4 relazioni di casi*" e a tal fine avete rilasciato il Vostro consenso informato scritto, sia per la partecipazione a tale Studio, sia per il necessario trattamento dei Vostri dati personali.

Tuttavia, conformemente alle Linee Guida del Garante per la Privacy (Deliberazione n. 52 del 24.07.2008) ed, in particolare, in considerazione dei dati trattati dal Promotore dello studio in oggetto, e fermo restando, comunque, il suo consenso a partecipare allo studio, è necessario integrare l'informativa che Vi è stata precedentemente rilasciata in merito alla tutela dei Vostri dati e raccogliere il Vostro consenso scritto per tale trattamento, da parte del Promotore dello studio e delle persone che per esso agiscono.

Titolari del trattamento e relative finalità

l'Istituto Scientifico IRCCS materno infantile Burlo Garofolo, che ha promosso e che coordina lo studio che Vi è stato illustrato in modo conforme alle norme di buona pratica clinica[1] e a quelle relative alla protezione dei dati personali[2], tratterrà i dati personali, in particolare quelli sulla salute del/la Vostro/a bambino/a, soltanto nella misura in cui sono indispensabili in relazione agli obiettivi dello studio, ed altri

---

1 A titolo esemplificativo e non esaustivo D.M. 15.7.1997, D. Lgs. 211/2003, D. Lgs. 200/2007 e successive modifiche e integrazioni).

2 D. Lgs. 196/2003 («Codice in materia di protezione dei dati personali»); «Linee Guida per i trattamenti di dati personali nell'ambito delle sperimentazioni cliniche dei medicinali» dell'Autorità garante per la protezione dei dati personali (Deliberazione n. 52 del 24/07/2008); «Autorizzazione generale al trattamento dei dati personali effettuato per scopi di ricerca scientifica» (Autorizzazione n. 9/ 11 dicembre 2014).

dati relativi agli stili di vita e il sesso, la data di nascita, l'età, il peso e l'altezza, la madrelingua, la storia medica, così come specificati precedentemente, esclusivamente in funzione della realizzazione dello studio.
A tal fine i dati indicati saranno raccolti dal centro di sperimentazione S.C. Otorinolaringoiatria ed Audiologia, conservati per 60 mesi dopo la fine dello studio; non verranno trasmessi a terzi soggetti. Il responsabile della conservazione e della gestione dei Vostri dati sarà la Dott.sa Eva Orzan, S.C. Otorinolaringoiatria e audiologia, IRCCS materno infantile "Burlo Garofolo" (tel. 040.3785.218, e-mail referente scientifico: amanda.saksida@burlo.trieste.it).
Il trattamento dei Vostri dati personali così come precedentemente precisato è indispensabile allo svolgimento dello studio medesimo: il rifiuto di conferirli non Vi consentirà di parteciparvi.

Natura dei dati

La Vostra identità e l'identità di Vostro/a figlio/a rimarrà sempre riservata e non sarà mai resa pubblica. Il Ricercatore che seguirà Vostro/a figlio/a nello studio lo/la identificherà con un codice numerico (ad esempio: xy0001) che non permette di risalire direttamente alla sua identità: i dati che Vi riguardano, raccolti nel corso dello studio, ad eccezione del Vostro nominativo e nominativo del seguirà Vostro/a figlio/a, saranno registrati, elaborati e conservati unitamente a tale codice, alla data di nascita, al sesso, al peso e alla statura, alla madrelingua, ed alle informazioni medico/cliniche raccolte ai fini dello studio. I dati saranno a cura della S.C. Otorinolaringoiatria e audiologia dell'IRCCS materno infantile "Burlo Garofolo". Soltanto lo sperimentatore e i soggetti autorizzati potranno collegare questo codice al Vostro nominativo, e solo quando indispensabile allo studio.

Modalità del trattamento

I dati, trattati mediante strumenti anche elettronici, saranno diffusi solo in forma rigorosamente anonima, ad esempio attraverso pubblicazioni scientifiche, statistiche e convegni scientifici. La partecipazione di Vostro/a figlio/a allo studio implica che, in conformità alla normativa sulle sperimentazioni cliniche dei medicinali, il personale del Promotore, il Comitato Etico e le Autorità Sanitarie italiane e straniere potranno accedere direttamente e conoscere i dati che La riguardano, contenuti anche nella Vostra documentazione clinica originale, con modalità tali da garantire la riservatezza della Vostra identità.
Inoltre, gli addetti al monitoraggio sono sottoposti a regole di condotta analoghe al segreto professionale, i professionisti sanitari sono soggetti al segreto professionale, i membri del Comitato Etico sono soggetti al segreto d'ufficio ed i soggetti terzi sono contrattualmente vincolati alla più assoluta confidenzialità e riservatezza.

Esercizio dei diritti

Potrete esercitare i diritti di cui all'art. 7 del Codice in materia di protezione dei dati personali - D. Lgs. 196, 30 giugno 2003 (es. accedere ai Suoi dati personali, integrarli, aggiornarli, rettificarli, opporsi al loro trattamento per motivi legittimi, ecc.) rivolgendosi direttamente al personale del Centro di sperimentazione, i cui nominativi sono riportati in calce a questa informativa. Ai medesimi

recapiti potrete richiedere l'elenco completo ed aggiornato dei Responsabili del trattamento dati eventualmente nominati.
Potrete interrompere in ogni momento e senza fornire alcuna giustificazione la Vostra partecipazione di Vostro/a figlio/a allo studio: in tal caso non saranno raccolti ulteriori dati che lo/a riguardano, ferma restando l'utilizzazione di quelli eventualmente già raccolti per determinare, senza alterarli, i risultati della ricerca.

Contatti

Se avete domande riguardo alla ricerca, ai vostri diritti come soggetto di ricerca o nell'eventualità di un danno correlato allo studio, vi preghiamo di contattare il medico dello studio o uno dei suoi collaboratori. Di seguito sono indicate tutte le persone che, presso questo centro/ospedale, svolgono attività di assistenza ai soggetti inclusi nello studio. Nel caso in cui questo elenco subisse variazioni nel corso dello studio, ne sarete informati verbalmente.

Vi ringraziamo per la Vostra disponibilità e la Vostra collaborazione

MODULO DI CONSENSO INFORMATO PER IL TRATTAMENTO DEI DATI PERSONALI

TITOLO DELLO STUDIO: **Valutazione delle capacità di comunicazione in bambini con primi IC bilaterali in ambienti clinici e domestici: 4 relazioni di casi**
CODICE DEL PROTOCOLLO: RC n. 17/17
PROMOTORE: IRCCS materno infantile "Burlo Garofolo"
STRUTTURA PRESSO LA QUALE SI SVOLGERÀ LO STUDIO: S.C. Otorinolaringoiatria e audiologia
SPERIMENTATORE RESPONSABILE: dott. Orzan Eva
CONTATTI per lo SPERIMENTATORE RESPONSABILE: Referente scientifico Dott.ssa Amanda Saksida
Tel: +386.40.843378 E-mail: amanda.saksida@burlo.trieste.it

PAZIENTE ____________________ _________________________ *(Nome e Cognome in stampatello)*
Numero identificativo assegnato al/alla paziente: ____
Io sottoscritta (*Nome e Cognome in stampatello*) ____________________________________,
nata __________________ il: __/__/_____ (madre)
Io sottoscritto (*Nome e Cognome in stampatello*) ____________________________________,
nato __________________ il: __/__/_____ (padre)
residente in _______________________________ Prov. __________ CAP ___________
Via _____________________n° _____Tel. ____________________
in proprio, ovvero nella qualità di RAPPRESENTANTE LEGALE / AMMINISTRATORE DI SOSTEGNO di
(*Nome e Cognome in stampatello*) ____________________ _________________________,
nato/a a ____ il ____________________________
residente in _______________________________ Prov. __________ CAP ___________
Via _____________________n° _____Tel. ____________________

Resta inteso che, nel caso in cui il paziente dovesse recuperare la capacità di intendere e di volere, sarà cura dello sperimentatore ottenere il consenso scritto da parte del paziente stesso (D.L. del 21-12-07, 6.1.2.5).In caso sia designato l'amministratore di sostegno, il medico sperimentatore avrà cura di verificare che l'ordinanza di affidamento da parte del giudice tutelare comprenda la tutela della salute dell'amministrato.

Dichiariamo:

- di aver preso visione dell'informativa scritta sopra riportata e di aver compreso le informazioni in essa contenute;
- di essere stato adeguatamente informato dei diritti esercitabili ai sensi dell'art. 7 del D. Lgs. 196 dd. 30.06.2003 ("Codice in materia di protezione dei dati personali").

Acconsentiamo: al trattamento dei miei dati personali e sanitari necessari allo svolgimento dello studio nei limiti e con le modalità indicate nell'informativa fornitami con il presente documento.

Dichiarazioni eventuali del/della paziente, domande aggiuntive o altri aspetti del consenso informato:

Nome e cognome per esteso del/la paziente o del legale rappresentante / amministratore di sostegno in stampatello:
____ (madre)
____ (padre)
Data: _______________
Firma del/la paziente o del rappresentante legale / amministratore di sostegno:
____ (madre)
____ (padre)
